# Deep Learning for Diagnosis of Paranasal Sinusitis Using Multi-View Radiographs

**DOI:** 10.3390/diagnostics11020250

**Published:** 2021-02-05

**Authors:** Yejin Jeon, Kyeorye Lee, Leonard Sunwoo, Dongjun Choi, Dong Yul Oh, Kyong Joon Lee, Youngjune Kim, Jeong-Whun Kim, Se Jin Cho, Sung Hyun Baik, Roh-eul Yoo, Yun Jung Bae, Byung Se Choi, Cheolkyu Jung, Jae Hyoung Kim

**Affiliations:** 1 Department of Radiology, Seoul National University Bundang Hospital, Seongnam 13620, Korea; snubhrad.yj@gmail.com (Y.J.); 92leekr@naver.com (K.L.); chzze4582@gmail.com (D.C.); dyoh1003@gmail.com (D.Y.O.); kjoon31@gmail.com (K.J.L.); sejinchorad@gmail.com (S.J.C.); mdshbaik@gmail.com (S.H.B.); bae729@gmail.com (Y.J.B.); byungse.choi@gmail.com (B.S.C.); jck0097@gmail.com (C.J.); jaehkim@snubh.org (J.H.K.); 2 Center for Artificial Intelligence in Healthcare, Seoul National Univeristy Bundang Hospital, Seongnam 13620, Korea; 3 Aerospace Medical Group, Air Force Education and Training Command, Jinju 52634, Korea; youngjune.kim.md@gmail.com; 4 Department of Otorhinolaryngology-Head and Neck Surgery, Seoul National University Bundang Hospital, Seongnam 13620, Korea; kimemails7@gmail.com; 5 Department of Radiology, Seoul National University Hospital, Seoul 03080, Korea; roheul7@gmail.com

**Keywords:** machine learning, deep learning, artificial intelligence, paranasal sinusitis, multi-view radiographs

## Abstract

Accurate image interpretation of Waters’ and Caldwell view radiographs used for sinusitis screening is challenging. Therefore, we developed a deep learning algorithm for diagnosing frontal, ethmoid, and maxillary sinusitis on both Waters’ and Caldwell views. The datasets were selected for the training and validation set (*n* = 1403, sinusitis% = 34.3%) and the test set (*n* = 132, sinusitis% = 29.5%) by temporal separation. The algorithm can simultaneously detect and classify each paranasal sinus using both Waters’ and Caldwell views without manual cropping. Single- and multi-view models were compared. Our proposed algorithm satisfactorily diagnosed frontal, ethmoid, and maxillary sinusitis on both Waters’ and Caldwell views (area under the curve (AUC), 0.71 (95% confidence interval, 0.62–0.80), 0.78 (0.72–0.85), and 0.88 (0.84–0.92), respectively). The one-sided DeLong’s test was used to compare the AUCs, and the Obuchowski–Rockette model was used to pool the AUCs of the radiologists. The algorithm yielded a higher AUC than radiologists for ethmoid and maxillary sinusitis (*p* = 0.012 and 0.013, respectively). The multi-view model also exhibited a higher AUC than the single Waters’ view model for maxillary sinusitis (*p* = 0.038). Therefore, our algorithm showed diagnostic performances comparable to radiologists and enhanced the value of radiography as a first-line imaging modality in assessing multiple sinusitis.

## 1. Introduction

Sinusitis is an inflammation of the membranes lining the paranasal sinus, which is one of the most frequently diagnosed diseases in the United States, affecting more than 15% of its population annually [1]. Sinusitis is diagnosed by evaluation of the patient’s history and physical examination, because clinical evaluation is usually sufficient to diagnose sinusitis in most cases and empirical treatments are cheap and safe. However, when symptoms are recurrent or persistent despite appropriate treatment, imaging of sinusitis may be required for further evaluation [2,3]. While CT is the imaging modality of choice for sinusitis, as it provides the highest overall anatomical detail of the paranasal sinuses, radiography is still widely used as an imaging modality when sinusitis is suspected because of its comparatively low cost, low radiation dose exposure, higher availability, and ease of examination [4,5]. 

The use of radiographic views such as Waters’ and Caldwell views is a conventional method for evaluation of the sinonasal area. Waters’ view, also known as the occipitomental view, is considered the best projection for evaluating maxillary sinuses. Meanwhile, the Caldwell view, also known as the occipitofrontal view, is applied chiefly for the evaluation of frontal and ethmoid sinuses [6,7]. However, the reliability of radiography in the evaluation of sinusitis is questionable [8,9]. The reported sensitivity is relatively low for all sinuses (25−41%) except for maxillary sinusitis (80%) [4]. Because adjacent bony shadows can overlap the sinuses, the interpretation of radiographs for sinusitis is difficult even for experienced radiologists, particularly when judging whether thickened mucous membrane is present [10].

Meanwhile, deep learning algorithms have recently begun to play an increasingly important role in analyzing medical images [11,12,13,14,15]. Such algorithms have been applied to various tasks such as lesion segmentation [11], detection [12], classification [13], reconstruction [14], and natural language processing [15]. In particular, recent studies [16,17] have demonstrated that deep learning algorithms can accurately classify maxillary sinusitis on Waters’ view. However, most of these studies of sinusitis based on deep learning have only focused on maxillary sinusitis with a single Waters’ view. Moreover, every image required manual cropping, which was time-consuming [16,17].

The purpose of our study was to develop a deep learning algorithm for the diagnosis of frontal, ethmoid, and maxillary sinusitis using both Waters’ and Caldwell views while avoiding the need for cropping and to compare its diagnostic performance with that of radiologists.

## 2. Materials and Methods

### 2.1. Dataset and Labeling

**Figure 1:**
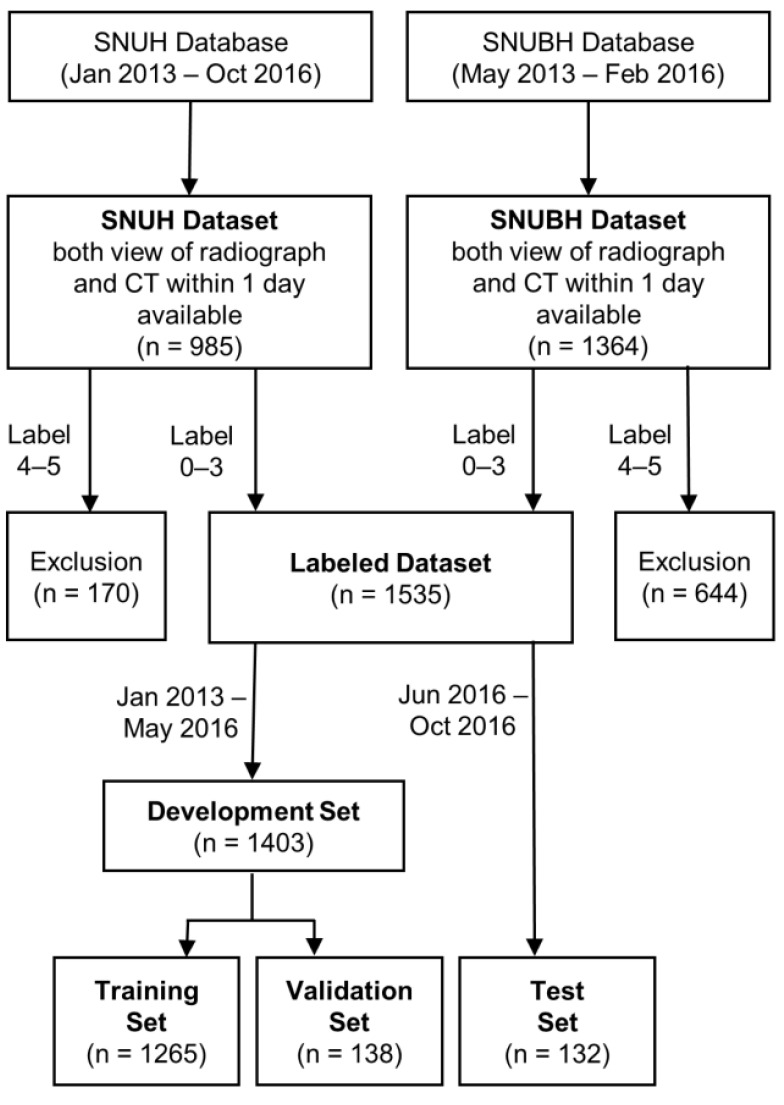
Flowchart of clinical datasets for training, validation, and test.

The data of 2349 consecutive patients older than 16 years who underwent Waters’- and Caldwell-view radiography and paranasal CT within a 1-day interval for suspected sinusitis were retrospectively retrieved from the databases of the Seoul National University Hospital (SNUH) between January 2013 and October 2016 and the Seoul National University Bundang Hospital (SNUBH) between May 2013 and February 2017 (Figure 1). As the pneumatization of the paranasal sinuses is completed by 15 years of age, only patients older than 16 years were included in this study [18,19]. Out of 3070 radiographs, 1152 (37.5%) from 1152 of 1535 patients (75.0%) in this study overlap with those included in our previous study [16]. While the prior study only covered the diagnosis of maxillary sinusitis on Waters’ view, the current study expands on the previous work by covering maxillary, frontal, and ethmoid sinusitis using both Waters’ and Caldwell views.

**Figure 2:**
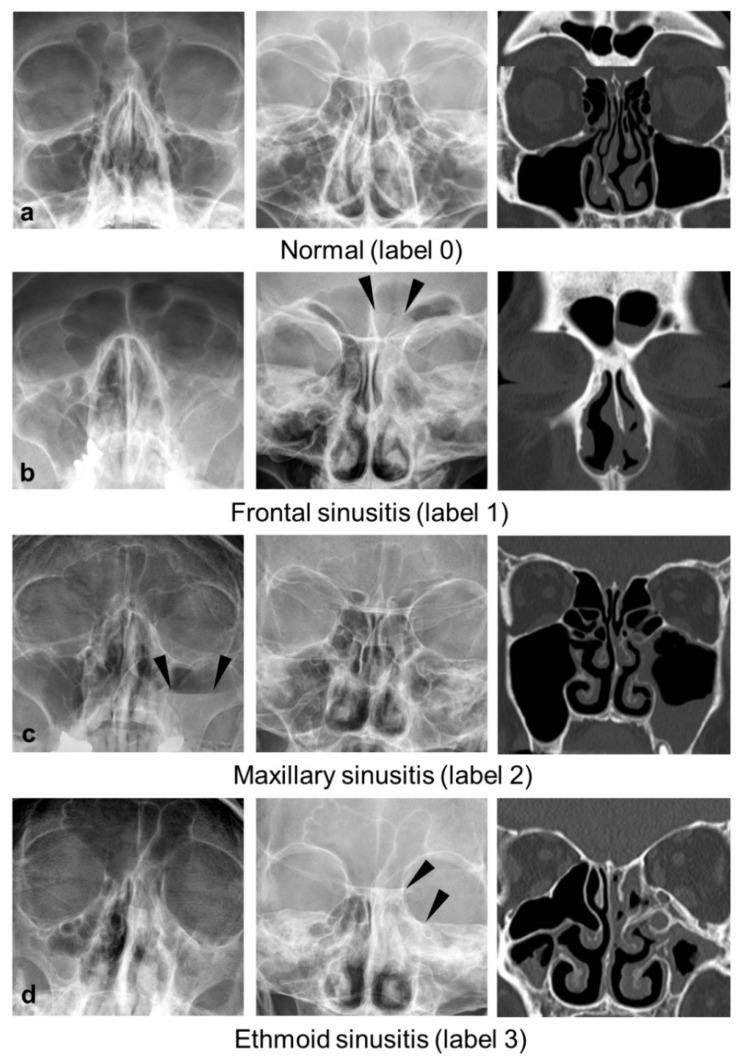
Representative cases with normal (label 0, **a**), frontal sinusitis (label 1, **b**), maxillary sinusitis (label 2, **c**), and ethmoid sinusitis (label 3, **d**) at each view (Waters’ view: left, Caldwell view: middle, corresponding coronal image of CT, right). For frontal (**b**) and ethmoid sinusitis (**d**), mucosal thickening (label 1) and total opacification (label 3) are not well visualized in Waters’ view, whereas Caldwell view provides the best projection for evaluation (arrowheads). In the case of maxillary sinusitis (**c**), Waters’ view provides a better view of the air-fluid level (label 2, arrowheads) than Caldwell view.

All studies were labeled by consensus of two radiologists (Y.J.B., an attending neuroradiologist with 10 years of experience, and Y.K., a board-certified radiologist with 4 years of experience) based on CT findings according to six types: 0, normal; 1, mucosal thickening (>4 mm for maxillary sinusitis, and >2 mm for frontal and ethmoid sinusitis); 2, air-fluid level; 3, total opacification; 4, interpretable but not belonging to any category (e.g., retention cyst); and 5, uninterpretable (e.g., poor image quality) (Figure 2). Because our model evaluates three sinuses bilaterally, six labels were recorded for each case.

After excluding 814 patients with labels 4 and 5, 1535 patients were finally included. Of these, the data of 132 patients on whom radiography was performed after June 2016 were used as the temporal test set. The data of the remaining 1403 patients were randomly split into 1265 datasets for training and 138 datasets for validation.

### 2.2. Network Architecture

**Figure 3:**
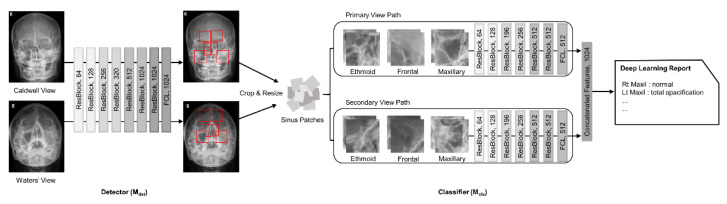
Overview of the proposed network architecture. The network consists of a detector (M_det_), which localizes sinuses with a bounding box, and a classifier (M_cls_), which classifies sinusitis with 4-leveled labels. It combines the information of both Caldwell and Waters’ views. For the modified model, which uses only the primary view (denoted as single primary view model), the network path of the second row is removed (vice versa for the single secondary view model, which uses only the secondary view).

Two deep convolutional neural networks were implemented with the use of TensorFlow (version 1.13.2) based on Python (version 3.7). Each convolutional neural network mainly comprised residual blocks aided by the squeeze-and-excitation module (Figure 3).

The first network (M_det_) acts as a detector for localizing each sinus area with bounding boxes. Network M_det_ consists of seven residual blocks, wherein the first five blocks are aided by the squeeze-and-excitation module. The second network (M_cls_) classifies each sinus patch proposed by M_det_ using four diagnostic labels. In particular, M_cls_ is a multi-view network merging enriched multi-angle information from patches of the primary and secondary views. Therefore, M_cls_ has two network paths: one for the primary view and the other for the secondary view. Each path consists of six residual blocks, followed by a feature concatenation layer.

### 2.3. Data Preprocessing

All radiographs were normalized for stable training, and random adjustment to contrast and brightness with random affine translation was applied for data augmentation. In particular, for the M_cls_ network, sinus patches were warped into 448 × 448 pixels via bilinear interpolation. Given that paranasal sinuses are basically symmetric, the left sinus patches were flipped horizontally to eliminate directional differences from the right ones.

### 2.4. Training Settings

L2 loss was used as the loss function of M_det_ for coordinate regression. For M_cls_, focal loss was used to alleviate the class imbalance problem. These two loss functions were minimized by the RMSProp optimizer with a learning rate of 0.001. M_det_ and M_cls_ were trained separately but were concatenated at the test stage to enable one-click prediction, which does not require any further operations. Moreover, corresponding class activation maps (CAMs) were extracted at the test stage to support and explain the prediction results of M_cls_ using the Grad-CAM method.

The main proposed model was constructed using a multi-view (MV) architecture, which simultaneously uses both Waters’ and Caldwell views. Moreover, to confirm the effect of each view, two modified models that focus on primary and secondary views separately were also trained (hereafter denoted as single primary view and single secondary view, respectively).

A single TITAN RTX GPU (Nvidia Corporation, Santa Clara, CA, USA) was used for accelerated training. To avoid overfitting, training was halted before the validation loss increased significantly.

### 2.5. Observer Study

To compare the performance of our algorithm with that of humans, we invited four radiologists as reviewers, and an observer study was conducted. For each case, the reviewers were asked to score each sinus using the 4-level diagnostic labels described above.

### 2.6. Statistical Analysis

To evaluate the performances of the proposed deep learning system and the four reviewers, we measured the sensitivity, specificity, positive predictive value, and areas under the receiver operating characteristic curve (AUC) for statistical metrics, and calculated the 95% CIs. In addition, Matthews correlation coefficient was calculated to measure the correlation between the prediction and ground truth label [20].

Before analysis, the labels were dichotomized into a normal set (label 0) and sinusitis set (labels 1–3). The AUCs were compared using the one-sided DeLong’s test [21]. To measure the sensitivity and specificity of the deep learning algorithm, three operating points at the optimal cutoff point, at a sensitivity of 90% (high sensitivity cutoff), and at a specificity of 90% (high specificity cutoff) were determined from the validation set. The optimal cutoff point was calculated by the index of the union method [22]. An adaptation of the single-treatment multiple-reader Obuchowski–Rockette model [23] was used to pool the AUCs of the radiologists.

Fleiss’ kappa statistics were used to calculate interobserver agreement among the radiologists. The level of agreement was interpreted as slight if κ was 0.01 to 0.20; fair, 0.21 to 0.40; moderate, 0.41 to 0.60; substantial, 0.61 to 0.80; and almost perfect, 0.81 to 1 [24]. We also investigated the agreement between the probability of sinusitis predicted by the algorithm and average diagnostic confidence levels rated by the four invited radiologists using Pearson’s correlation coefficient.

All statistical analyses were performed by using the statistical software R (version 3.6.2, R Foundation for Statistical Computing, Vienna, Austria). In particular, “RJafroc: Artificial Intelligence Systems and Observer Performance” (https://cran.r-project.org/web/packages/RJafroc) library was used to perform single-treatment multiple-reader pooling to calculate overall sensitivity and specificity. A *p*-value of <0.05 was considered significant. To account for multiple comparisons between the radiologists and the deep learning algorithm, a Bonferroni correction was applied to each sinus using an adjusted α-level of 0.013 (0.05/4) [25].

## 3. Results

### 3.1. Patient Demographics

Table 1 summarizes the patient baseline characteristics. The training and validation sets included 735 men (52.4%) and 668 women (47.6%), and the test set included 57 men (43.2%) and 75 women (56.8%). The mean age was 50 ± 19 years for the training and validation sets and 54 ± 17 years for the test set.

### 3.2. Performance Comparison of Deep Learning Models

Table 2 summarizes the performance of the deep learning algorithm. The AUCs of the MV-based deep learning algorithm were higher than those of the single secondary view for maxillary and ethmoid sinusitis, and they were comparable with those of the single primary view for frontal, ethmoid, and maxillary sinusitis and the single secondary view for frontal sinusitis.

In particular, the MV model exhibited better performance than the single primary view and single secondary view models for maxillary sinusitis (*p* = 0.038 and <0.001, respectively). The MV model also outperformed the single secondary view regarding ethmoid sinusitis (*p* = 0.004).

### 3.3. Performance Comparison of Multi-View Model with Radiologists

**Figure 4:**
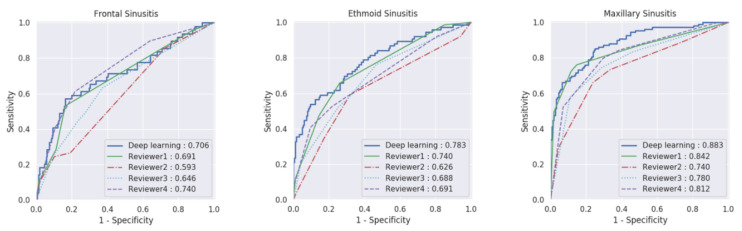
Receiver operating characteristic (ROC) curves and the area under the ROC curves (AUCs) of the proposed multi-view model and reviewers in the observer performance study.

The MV model demonstrated an AUC greater than that of the radiologists for maxillary and ethmoid sinusitis (*p* = 0.013 and *p* = 0.012, respectively) (Figure 4). The AUC range of the radiologists was 0.74–0.84 for maxillary sinusitis, 0.63–0.74 for ethmoid sinusitis, and 0.59–0.73 for frontal sinusitis. For maxillary and ethmoid sinusitis, the MV model showed a higher AUC than that of three of the four radiologists (maxillary, *p* < 0.001, 0.002, and 0.016; ethmoid, *p* < 0.001, 0.024, and 0.028, respectively). Regarding frontal sinusitis, the AUC of the MV model was higher than that of one of the four radiologists (*p* = 0.032).

Table 3 lists the sensitivities and specificities of our algorithm and of radiologists. The sensitivity range for the radiologists was 72.5–84.4% for maxillary sinusitis, 50.0–61.8% for ethmoid sinusitis, and 26.5–49.0% for frontal sinusitis. The specificity range of the radiologists was 65.8–87.1% for maxillary sinusitis, 66.0–82.4% for ethmoid sinusitis, and 74.0–85.6% for frontal sinusitis. Overall, the MV model exhibited superior sensitivity and specificity relative to the radiologists’ average scores.

The interobserver agreement (κ) values among the invited radiologists for diagnosing maxillary, ethmoid, and frontal sinusitis were 0.49, 0.33, and 0.24, respectively. Scatter plots of the average of radiologists’ diagnostic confidence levels versus the probability of sinusitis predicted by the deep learning algorithm for each sinus are shown in Supplementary Figure S1. The correlation coefficients between the predicted probability of the algorithm and confidence levels of radiologists were 0.81, 0.57, and 0.51 for maxillary, ethmoid, and frontal sinus, respectively. Confusion matrices of predicted and ground truth labels in the external test set are shown in Supplementary Figure S2.

**Figure 5:**
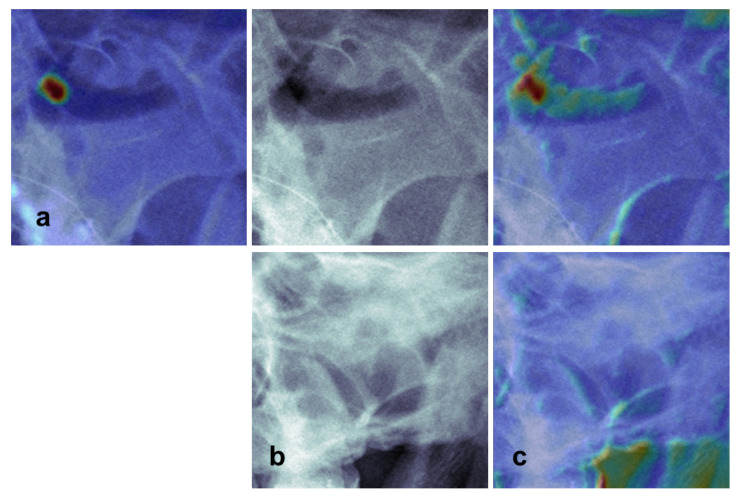
Comparison of the class activation maps (CAMs) of the single primary view and multi-view model. (**a**) CAM of the single primary view model (Waters’ view). (**b**) Original radiographs showing maxillary sinusitis with air-fluid level (upper row, Waters’ view; lower row, Caldwell view) (**c**), CAM of the multi-view model (upper row, Waters’ view; lower row, Caldwell view). Single primary view model (**a**) misclassified as mucosal thickening because the area above the horizontal fluid line is not recognized. In the case of the multi-view model (**c**), the prediction is correct as the activated area is more expanded on Waters’ view (upper row), while no clear activation is found in the sinus area on the secondary view (lower row).

The representative images with the CAMs of the single primary view and MV models are shown in Figure 5. We note that the single primary view model misclassified the image with the air-fluid level (label 2) as mucosal thickening (label 1). However, the MV model recognized the air-fluid level and correctly classified the image under label 2. The activated area in the CAM was expanded for the MV model because no clear area in the maxillary sinus was detected on the secondary view. Representative images of the primary view with class activation mapping in normal and sinusitis cases are shown in Supplementary Figure S3. Class activation mapping showed that activation primarily occurred along the bony wall of the sinus and the clear area within the sinus. Additional examples showing false-positive and false-negative cases are shown in Supplementary Figure S4. To validate the results, we additionally trained the model with the development dataset using 5-fold cross-validation. The result of the cross-validation model is presented in Supplementary Table S1 and Supplementary Figure S5.

## 4. Discussion

In this study, we developed a deep learning algorithm for diagnosing multiple sites of sinusitis on radiographs. Our proposed algorithm detects and classifies each sinus simultaneously and therefore does not require manual cropping as a preprocessing step. It can accurately diagnose multiple sites of sinusitis using both Waters’ and Caldwell views as input images. We found that the multi-view model outperforms the single primary and secondary view models, particularly for maxillary sinusitis. The proposed algorithm also outperforms results obtained by radiologists, particularly for ethmoid and maxillary sinusitis.

The diagnostic performance of deep learning in classifying maxillary sinusitis was comparable to the two previous studies [16,17], with AUC ranging from 0.88–0.93 [16] and 0.88–0.94 [17], respectively. While these studies [16,17] have only evaluated maxillary sinusitis using Waters’ view, we additionally evaluated the diagnostic performance for frontal and ethmoid sinusitis using multi-view radiographs. Although maxillary sinusitis is more common than frontal or ethmoid sinusitis, accurate diagnosis of frontal and ethmoid sinusitis is also important. The local inflammation or anatomic obstruction of the ostiomeatal complex interferes the mucociliary clearance and leads to sinusitis development [26]. In particular, the anterior ethmoid, which is located close to the ostiomeatal complex, plays an important role in the pathophysiology of sinusitis [27]. Furthermore, complications of frontal sinusitis can become life-threatening by involving intracranial structures [28].

Unlike previous approaches requiring cropping of sinus patches from radiographs [16,17], we designed a model comprising a detector (M_det_) and a classifier (M_cls_). The M_det_ network eliminated the manual cropping requirement through automatic detection of the sinus area. Meanwhile, to simultaneously use multi-view information from two images, M_cls_ was designed to concatenate two multi-view features. Among the several studies that used multi-view models [29,30,31], we adapted the model proposed by Kim et al. [29], which merges the features of the three views of the shoulder radiograph at the fully connected layer with encoded clinical information.

The interpretation of sinusitis on radiographs relies on the detection of the bony wall of each sinus and the subsequent assessment of mucosal thickening or accumulation of mucopus. The sclerotic/erosive bone change or the atelectatic change of a sinus may serve as an ancillary finding. Each of the skull bones casts multiple superimposed shadows [6], which are subject to large changes with small changes of the head position. Therefore, each paranasal radiograph offers its own best visualized structures by minimizing the overlapped shadows at different orientations. Our study demonstrated that the performance of the MV model was comparable with or modestly superior to that of the single primary view model, indicating that the contribution of the secondary view is small compared to that of the primary view.

Although acute sinusitis is routinely diagnosed on clinical grounds, the accuracy of clinical diagnosis for sinusitis remains controversial. One study [32] reported that 34.7% of patients diagnosed with sinusitis had negative results on CT. A meta-analysis of six studies revealed that radiographs show moderate sensitivity (73%) and specificity (80%) when compared with these parameters for sinus puncture, and the analysis suggested that clinical criteria may exhibit a diagnostic accuracy similar to that of the radiographs [33,34]. Therefore, correlation of both clinical and radiographic findings is important.

In this study, we determined three cutoff values in the validation set following previous studies [16,35]: the optimal cutoff and 90% high-sensitivity and high-specificity cutoffs. Considering that radiography is primarily used for the screening of paranasal sinusitis, a high-sensitivity operating point should be selected. Although many attempts have been made to reduce the radiation dose of the paranasal sinus CT [36], the estimated effective dose is still higher than that of radiography, which is important owing to the increased risk of leukemia and solid cancer [37,38]. Using our MV model may enhance the value of radiography as a first-line imaging modality in assessing multiple sinusitis with low radiation dose, low cost, higher availability, and ease of examination. The observers showed moderate, fair, and fair interobserver agreements for maxillary, ethmoid, and frontal sinuses, respectively. The relatively low agreement levels for ethmoid and frontal sinuses demonstrate the need for a decision support system, such as our proposed algorithm. In particular, the probability of sinusitis predicted by the proposed algorithm and the radiologists’ confidence levels generally correlated well, with the correlation coefficients ranging from 0.51 to 0.81. Therefore, the proposed algorithm may aid in overcoming the intrinsic low interobserver and intraobserver agreements in radiographs [39] and improving the diagnostic consistency.

This study has several limitations. First, the data size was relatively small because we opted to include patients for whom paranasal radiography and CT were performed within a 1-day interval. In particular, data imbalance of label 2 in the frontal and maxillary sinusitis dataset exists. However, we believe that the influence of shortage in label 2 cases to the overall performance is limited because the final task was to predict whether there is sinusitis or not in each sinus (i.e., dichotomized to label 0 vs. 1–3). Second, the reference standard used for this study was only CT and did not consider clinical findings. However, the diagnostic criteria for sinusitis used in this study can be confidently determined on CT, and this approach results in high reliability, particularly for frontal and ethmoid sinusitis. Finally, this was a retrospective study and therefore does not precisely represent real-world scenarios. Further studies are necessary to determine the clinical usefulness of our algorithm in a prospective setting.

## 5. Conclusions

Our deep learning algorithm was able to reliably assess frontal, ethmoid, and maxillary sinusitis on Waters’ and Caldwell view radiographs, and the algorithm outperformed the radiologists for ethmoid and maxillary sinusitis.

## Figures and Tables

**Table 1:** Patient characteristics and label distribution of training, validation, and temporal test set.

Category	Training Set	Validation Set	Temporal Test Set	Total
Number of Patients	1265	138	132	1535
Age (y) †	50 ± 19	49 ± 18	54 ± 17	51 ± 19
Sex				
Men	659 (52.1)	76 (55.1)	57 (43.2)	792 (51.6)
Women	606 (47.9)	62 (44.9)	75 (56.8)	743 (48.4)
Frontal Sinusitis ‡				
0—normal	2029 (80.2)	212 (76.8)	215 (81.4)	2456 (80.0)
1—mucosal thickening > 2 mm	286 (11.3)	38 (13.8)	37 (14.0)	361 (11.8)
2—air-fluid level	3 (0.1)	1 (0.4)	0 (0.0)	4 (0.1)
3—total opacification	212 (8.4)	25 (9.1)	12 (4.5)	249 (8.1)
Ethmoid Sinusitis ‡				
0—normal	1589 (62.8)	178 (64.5)	188 (71.2)	1955 (63.7)
1—mucosal thickening > 2 mm	171 (6.8)	14 (5.1)	20 (7.6)	205 (6.7)
2—air-fluid level	184 (7.3)	18 (6.5)	21 (8.0)	223 (7.3)
3—total opacification	586 (23.2)	66 (23.9)	35 (13.3)	687 (22.4)
Maxillary Sinusitis ‡				
0—normal	1375 (54.3)	149 (54.0)	155 (58.7)	1679 (54.7)
1—mucosal thickening > 4 mm	738 (29.2)	77 (27.9)	87 (33.0)	902 (29.4)
2—air-fluid level	145 (5.7)	22 (8.0)	5 (1.9)	172 (5.6)
3—total opacification	272 (10.8)	28 (10.1)	17 (6.4)	317 (10.3)

Note—Except where indicated, data are the numbers of patients, with percentages in parentheses. † Data are the means ± standard deviation. ‡ Data are the numbers of sinuses, thus doubled by the number of patients.

**Table 2:** Performance of deep learning model in diagnosing multiple sinusitis at each view.

	Single Primary View	Single Secondary View	Multi-View
Frontal Sinusitis			
AUC	0.72 (0.63–0.80)	0.72 (0.65–0.80)	0.71 (0.62–0.80)
*p*-Value *	0.645	0.685	
Ethmoid Sinusitis			
AUC	0.79 (0.73–0.85)	0.70 (0.63–0.77)	0.78 (0.72–0.85)
*p*-Value *	0.566	0.004	
Maxillary Sinusitis			
AUC	0.86 (0.81–0.90)	0.76 (0.70–0.81)	0.88 (0.84–0.92)
*p*-Value *	0.038	<0.001	

Note—The numbers in parentheses are 95% confidence intervals. AUC = Area under the receiver operating characteristic curve. * Compared with multi-view. *p*-values are calculated with one-sided DeLong’s test.

**Table 3:** Performance of deep learning model and radiologists in diagnosing multiple sinusitis.

**Frontal Sinusitis**	**Sensitivity**	**Specificity**	**PPV**	**MCC**	**AUC**	***p*-Value †**
Deep learning algorithm					0.71 (0.62–0.80)	
Optimal Cutoff †	57.1 (42.2–71.2)	82.8 (77.1–87.6)	43.1 (30.8–56.0)	0.36 (0.25–0.46)		
Cutoff for High Sensitivity	71.4 (56.7–83.4)	48.8 (42.0–55.7)	24.1 (17.4–31.9)	0.16 (0.04–0.27)		
Cutoff for High Specificity	40.8 (27.0–55.8)	90.2 (85.5–93.9)	48.8 (32.9–64.9)	0.33 (0.22–0.44)		
Radiologist						
1	49.0 (34.4–63.7)	85.6 (80.2–90.0)	43.6 (33.4–54.4)	0.33 (0.22–0.43)	0.69 (0.61–0.77)	0.402
2	26.5 (14.9–41.1)	85.1 (79.6–89.6)	28.9 (18.8–41.7)	0.12 (0.00–0.24)	0.59 (0.51–0.67)	0.032
3	46.9 (32.5–61.7)	74.0 (67.5–79.7)	29.1 (22.0–37.4)	0.18 (0.06–0.29)	0.64 (0.56–0.73)	0.165
4	49.0 (34.4–63.7)	84.7 (79.1–89.2)	42.1 (32.2–52.6)	0.32 (0.20–0.42)	0.73 (0.66–0.82)	0.711
Overall ‡	42.9	82.4			0.66 (0.56–0.78)	0.477
**Ethmoid Sinusitis**	**Sensitivity**	**Specificity**	**PPV**	**MCC**	**AUC**	***p*-Value †**
Deep learning algorithm					0.78 (0.72–0.85)	
Optimal Cutoff †	59.2 (47.3–70.4)	83.5 (77.4–88.5)	59.2 (47.3–70.4)	0.43 (0.32–0.52)		
Cutoff for High Sensitivity	78.9 (68.1–87.5)	59.0 (51.7–66.1)	43.8 (35.3–52.5)	0.34 (0.23–0.45)		
Cutoff for High Specificity	38.2 (27.2–50.0)	95.2 (91.1–97.8)	76.3 (59.8–88.6)	0.43 (0.33–0.52)		
Radiologist						
1	61.8 (50.0–72.8)	78.2 (71.6–83.9)	53.4 (45.3–61.3)	0.38 (0.28–0.48)	0.74 (0.68–0.80)	0.170
2	51.3 (39.6–63.0)	75.0 (68.2–81.0)	45.4 (37.4–53.6)	0.25 (0.14–0.36)	0.63 (0.55–0.70)	**<0.001** *
3	61.8 (50.0–72.8)	66.0 (58.7–72.7)	42.3 (36.0–48.9)	0.25 (0.14–0.36)	0.69 (0.62–0.76)	0.024
4	50.0 (38.3–61.7)	82.4 (76.2–87.6)	53.5 (44.0–62.8)	0.33 (0.22–0.43)	0.69 (0.62–0.76)	0.028
Overall ‡	56.2	75.4			0.69 (0.61–0.76)	0.012 *
**Maxillary Sinusitis**	**Sensitivity**	**Specificity**	**PPV**	**MCC**	**AUC**	***p*-Value †**
Deep learning algorithm					0.88 (0.84–0.92)	
Optimal Cutoff †	80.3 (69.5–88.5)	71.8 (64.8–78.1)	78.6 (69.1–86.2)	0.58 (0.50–0.66)		
Cutoff for High Sensitivity	85.3 (77.3–91.4)	72.9 (65.2–79.7)	68.9 (60.4–76.6)	0.57 (0.49–0.65)		
Cutoff for High Specificity	63.3 (53.5–72.3)	93.5 (88.5–96.9)	87.3 (78.0–93.8)	0.61 (0.53–0.68)		
Radiologist						
1	75.2 (66.0–83.0)	87.1 (80.8–91.9)	80.4 (72.9–86.2)	0.63 (0.55–0.70)	0.84 (0.79–0.89)	0.112
2	72.5 (63.1–80.6)	69.7 (61.8–76.8)	62.7 (56.3–68.7)	0.42 (0.31–0.51)	0.74 (0.67–0.80)	**<0.001** *
3	74.3 (65.1–82.2)	70.3 (62.5–77.4)	63.8 (57.4–69.7)	0.44 (0.34–0.53)	0.78 (0.72–0.84)	**0.002** *
4	84.4 (76.2–90.6)	65.8 (57.8–73.2)	63.5 (57.9–68.7)	0.50 (0.40–0.58)	0.81 (0.76–0.86)	0.016
Overall ‡	76.6	73.2			0.79 (0.73–0.86)	0.013 *

Note—Asterisk (*) indicates significant results. Bold indicates correlation is significant at alpha level corrected by Bonferroni method. Numbers in parentheses are 95% confidence intervals (CIs). AUC = area under the receiver operating characteristic curve, PPV = positive predictive value, MCC = Matthews correlation coefficient. Sensitivity and specificity presented in percentage. † Optimal cutoffs and *p*-values are calculated with index of union and one-sided DeLong’s test. ‡ Overall sensitivity and specificity are represented with arithmetic mean value to roughly indicate the performances of the reviewer group. Overall AUC with CI is calculated using the pooling method of the single-treatment multiple-reader Obuchowski–Rockette model to compare it with the AUC obtained by the standalone deep learning algorithm.

## Data Availability

The datasets generated during and/or analyzed during the current study are available from the corresponding author on reasonable request, but is subject to the permission of the Institutional Review Boards of the participating institutions.

## Supplementary Materials

The following are available online at https://www.mdpi.com/2075-4418/11/2/250/s1, Figure S1: Scatter plots of the average of radiologists’ diagnostic confidence levels versus the probability of sinusitis predicted by the deep learning algorithm for each sinus; Figure S2: Confusion matrices of predicted and ground truth labels in external test sets; Figure S3: Representative cases with the primary view (Waters’ view in this case) with superimposed heatmap using class activation mapping; Figure S4: Examples of false-positive (a) and false-negative (b) cases; Figure S5: Confusion matrices of predicted and ground truth labels using 5-fold cross-validation model; Table S1: Performance of deep learning using 5-fold cross-validation model in diagnosing multiple sinusitis.
