# Supplementary material for: Deep Learning for Diagnosis of Paranasal Sinusitis Using Multi-View Radiographs
Source: Diagnostics (Basel). 2021 Feb 5;11(2):250. doi: 10.3390/diagnostics11020250 (PMC7914751; doi:10.3390/diagnostics11020250)
Supplement: Supplementary file 1 [file diagnostics-11-00250-s001.zip › diagnostics-1083409-supplementary.pdf]

Supplementary Materials

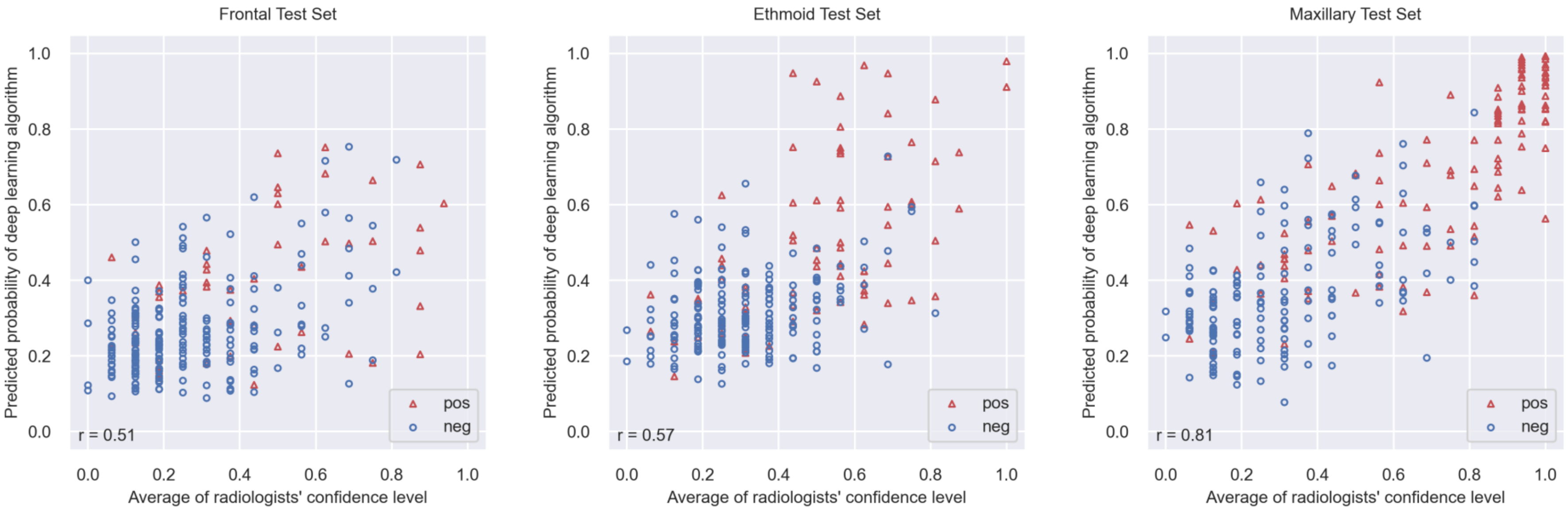

Supplementary Figure S1. Scatter plots of the average of radiologists' diagnostic confidence levels versus the probability of sinusitis predicted by the deep learning algorithm for each sinus. The blue circles represent normal cases, and the red triangles represent the cases with each type of sinusitis. The probability of sinusitis predicted by the proposed algorithm and the radiologists' confidence levels generally correlated well, particularly for the maxillary sinus (Pearson's correlation coefficient r = 0.81, 0.57, and 0.51, for maxillary, ethmoid, and frontal sinus, respectively).

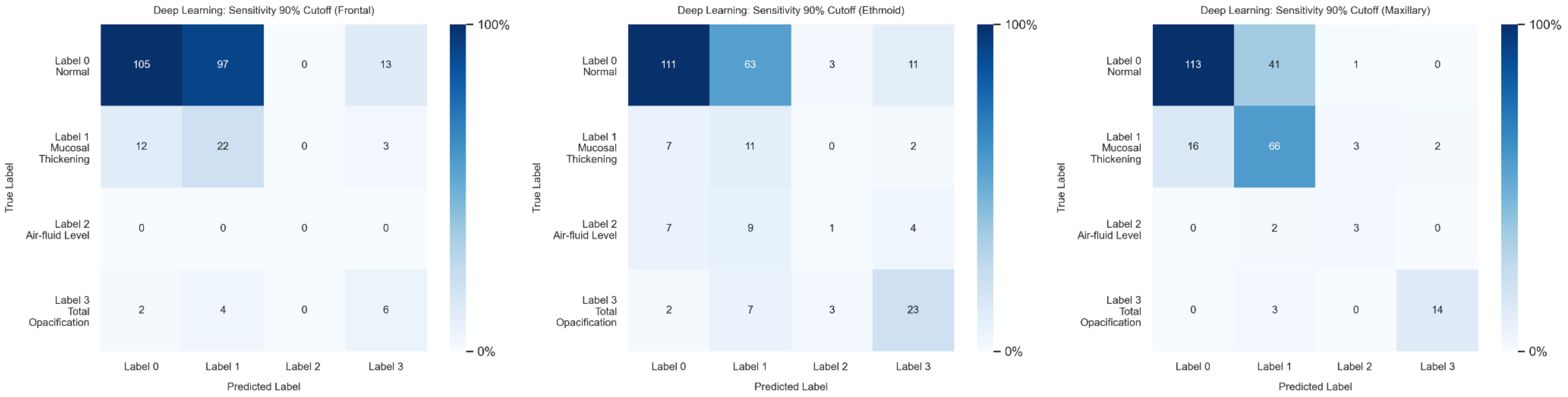

Supplementary Figure S2. Confusion matrices of predicted and ground truth labels in external test sets. Element (i, j) of each confusion matrix represents the predicted probability of predicting label j given that the ground truth was label i.

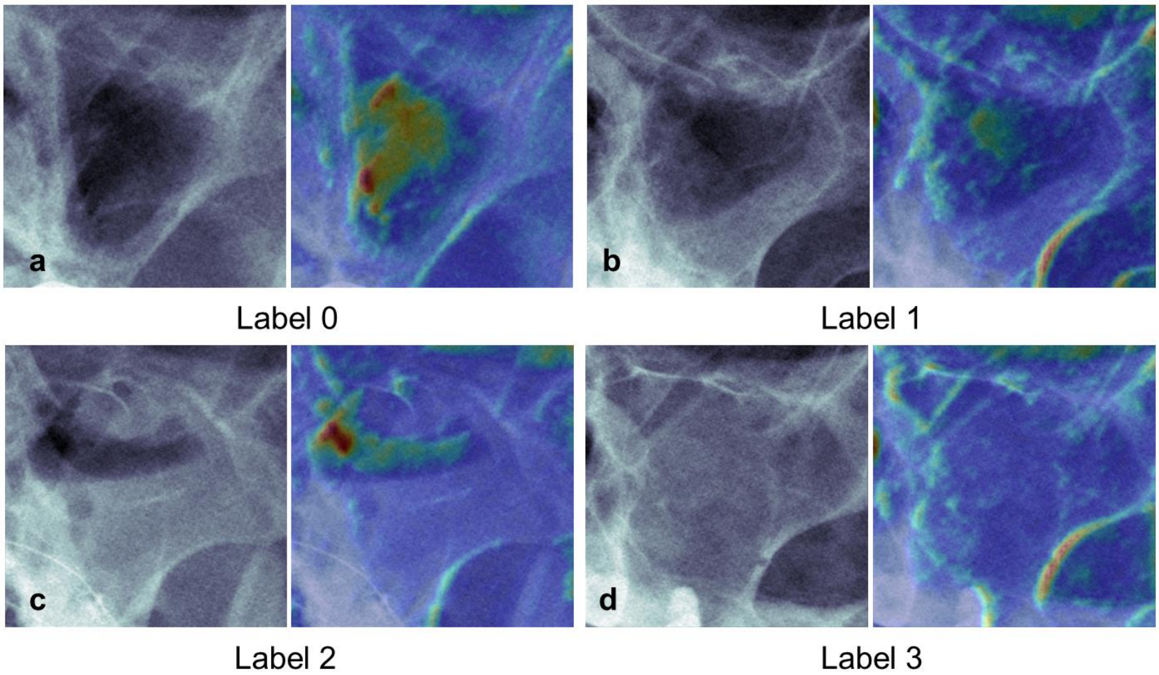

Supplementary Figure S3. Representative cases with the primary view (Waters' view in this case) with superimposed heatmap using class activation mapping. The model mainly searches the cavitary portion within the sinus and the along the bony sinus wall. In the normal case (a), a relatively large air-containing cavity area is activated, compared to the case with mucosal thickening (b) and the case with air-fluid level (c). In case of total opacification (d), no activation occurred within the sinus area at all.

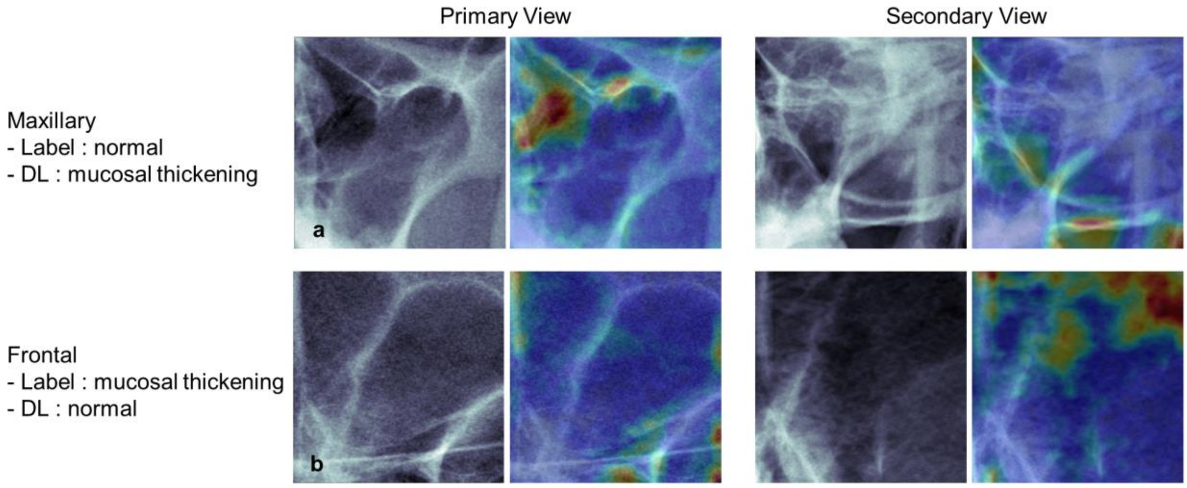

Supplementary Figure S4. Examples of false-positive (a) and false-negative (b) cases.

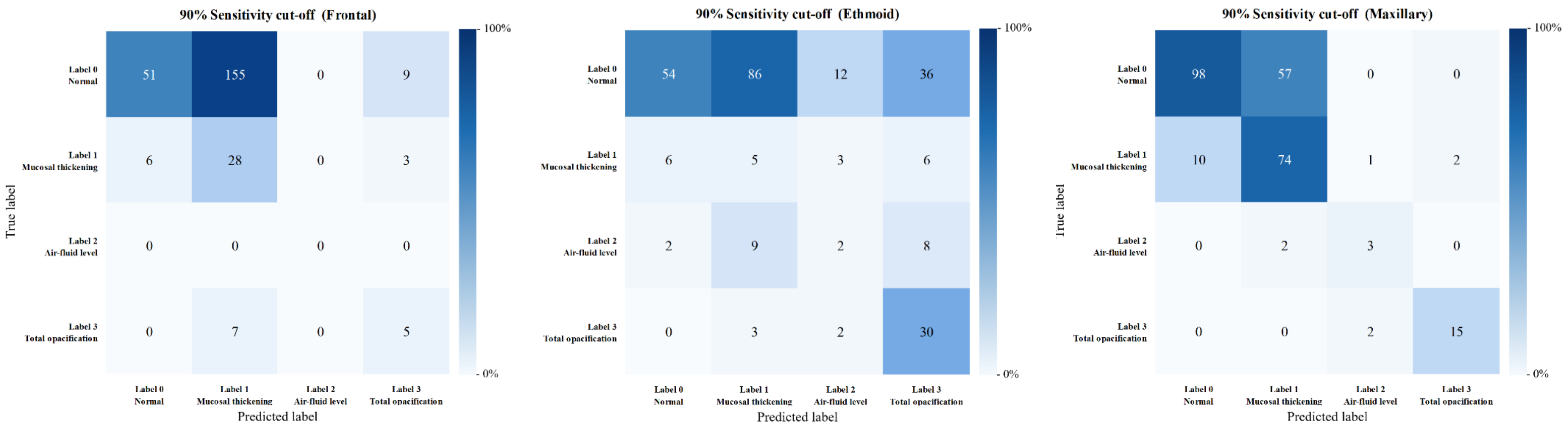

Supplementary Figure S5. Confusion matrices of predicted and ground truth labels using 5-fold cross-validation model. Element (i, j) of each confusion matrix represents the predicted probability of predicting label j given that the ground truth was label i.

	Frontal Sinusitis	Ethmoid Sinusitis	Maxillary Sinusitis
	AUC	AUC	AUC
CV1	0.73	0.77	0.88
CV2	0.73	0.79	0.89
CV3	0.73	0.77	0.89
CV4	0.73	0.75	0.86
CV5	0.71	0.82	0.87
Overall (95% CI)	0.75 (0.66-0.83)	0.80 (0.74-0.87)	0.89 (0.85-0.93)

Supplementary Table S1. Performance of deep learning using 5-fold cross-validation model in diagnosing multiple sinusitis.
